# Extracting consistent knowledge from highly inconsistent cancer gene data sources

**DOI:** 10.1186/1471-2105-11-76

**Published:** 2010-02-05

**Authors:** Xue Gong, Ruihong Wu, Yuannv Zhang, Wenyuan Zhao, Lixin Cheng, Yunyan Gu, Lin Zhang, Jing Wang, Jing Zhu, Zheng Guo

**Affiliations:** 1 College of Bioinformatics Science and Technology, Harbin Medical University, Harbin 150086, China; 2 Bioinformatics Centre, School of Life Science, University of Electronic Science and Technology of China, Chengdu, 610054, China

## Abstract

**Background:**

Hundreds of genes that are causally implicated in oncogenesis have been found and collected in various databases. For efficient application of these abundant but diverse data sources, it is of fundamental importance to evaluate their consistency.

**Results:**

First, we showed that the lists of cancer genes from some major data sources were highly inconsistent in terms of overlapping genes. In particular, most cancer genes accumulated in previous small-scale studies could not be rediscovered in current high-throughput genome screening studies. Then, based on a metric proposed in this study, we showed that most cancer gene lists from different data sources were highly functionally consistent. Finally, we extracted functionally consistent cancer genes from various data sources and collected them in our database F-Census.

**Conclusions:**

Although they have very low gene overlapping, most cancer gene data sources are highly consistent at the functional level, which indicates that they can separately capture partial genes in a few key pathways associated with cancer. Our results suggest that the sample sizes currently used for cancer studies might be inadequate for consistently capturing individual cancer genes, but could be sufficient for finding a number of cancer genes that could represent functionally most cancer genes. The F-Census database provides biologists with a useful tool for browsing and extracting functionally consistent cancer genes from various data sources.

## Background

Cancer is an extremely heterogeneous disease that is induced by mutations or other alterations in many genes [1,2]. Identification of genes that are causally implicated in oncogenesis is of basic importance for predicting novel cancer genes [3-6], and studying their evolutionary conservation [6,7], biological network features [4,8] and functions [9-11]. It can also provide valuable biomarkers for cancer diagnosis and drug development [2,11]. Until now, hundreds of cancer genes that have been found in small-scale experiments have been collected in various databases such as Cancer Gene Census (CGC) [12], Online Mendelian Inheritance in Man (OMIM) [13], and many others [14-17]. Recently, by high-throughput somatic mutational screening of cancer genomes [18-24], hundreds of new cancer genes that carry driver mutations are being identified rapidly. These increasingly abundant data provide us with an excellent opportunity to understand the underlying complex mechanisms of oncogenesis.

Nevertheless, we face new challenges to interpret and apply these abundant yet diverse data sources efficiently. In particular, it is important to evaluate the consistency and reliability of the information from different data sources. In this work, we analyzed six lists of cancer genes separately from six major databases [12-17] and two lists of candidate cancer genes identified by two types of high-throughput techniques [19,20,22,23,25,26]. First, we showed that these gene lists were highly inconsistent in terms of overlapping genes, which reflected partially their various types of cancer and mutations. In particular, most cancer genes accumulated in small-scale experiments could not be reproduced in current high-throughput mutational screening of cancer genomes, even when comparing cancer type-specific genes. This suggests that the sample sizes used in the small-scale studies or high-throughput genome screening might have been too small to capture consistently genes that are causally related to cancers with extremely heterogeneous genetic mechanisms.

On the other hand, various gene lists might capture separately different genes in a few functional pathways that are related to human cancer [1,18,20,21,27-29]. Based on protein-protein interaction (PPI) data, we introduced the POGF (Percentage of Overlapping Genes Functionally related) metric to evaluate the functional consistency of gene lists, and found that most of them were actually highly functionally consistent. Specifically, most cancer genes accumulated in previous small-scale studies could be functionally reproduced in current high-throughput studies.

The CGC database is the most widely utilized cancer gene data source [3-6,8,11,22,23], therefore, we used it as a benchmark for evaluating and selecting functionally consistent cancer genes from other data sources. We found that the selected genes were more significantly enriched in cancer pathways than the rest of the genes. Finally, we developed the database F-Census for collecting functionally consistent cancer genes from various data sources http://bioinfo.hrbmu.edu.cn/fcensus/.

## Methods

### Cancer gene lists

We analyzed six databases of cancer genes whose alterations might play causative roles in carcinogenesis (Table 1). We also analyzed a list of 491 cancer genes provided by mutational screening in cancer genomes for four cancer types [19,20,22,23] and another list of 645 cancer genes identified by retroviral insertional mutagenesis screening [25,26].

**Table 1:** The eight cancer gene lists analyzed in this paper

Short names	Full names and URLs	References	No. of genes
CGC	Cancer Gene Census http://www.sanger.ac.uk/genetics/CGP/Census/	[12]	377*(328**)
OMIM^a^	Online Mendelian Inheritance in Man http://www.ncbi.nlm.nih.gov/sites/entrez?db=omim	[13]	244 (217)
Reviews^b^	CancerGenes database http://cbio.mskcc.org/CancerGenes/Select.action	[14,28,50]	289(261)
AGCOH	Atlas of Genetics and Cytogenetics in Oncology and Haematology http://atlasgeneticsoncology.org/	[17]	727(619)
TGDBs	The Tumor Gene Family of Databases http://www.tumor-gene.org/Oral/oral.html	[16,51,52]	314(295)
TSGDB	Tumor suppressor gene database http://www.cise.ufl.edu/~yy1/HTML-TSGDB/Homepage.html	[15]	148(109)
H-list	Candidate cancer genes provided by genome mutation scans	[19,20,22,23]	491(316)
R-list	Candidate cancer genes identified by retroviral insertional mutagenesis screens http://RTCGD.ncifcrf.gov http://mutapedia.nki.nl	[25,26]	646(496)
Total			2105 (1594)

Note: *the numbers of genes compiled in the original datasets; **the numbers of genes with PPI data. ^a ^cancer genes were extracted from OMIM as did in [53]. ^b ^cancer genes from two Reviews which are collected in the CancerGenes database [28,50].

### PPI and Gene Ontology (GO) data

The PPI data were derived from the Human Protein Reference Database (HPRD, release 7) [30], which contains 34 998 interactions that involve 9303 proteins after removing self-interactions, including 13 080 interactions between 6311 proteins derived from high-throughput yeast two-hybrid experiments. The GO annotation data [31] were downloaded on September 1, 2008.

### Evaluating the consistency of gene lists by POG scores

The POG (Percentage of Overlapping Genes) metric was used to evaluate the consistency of two gene lists [32-34]. If list 1 with length *l*_1 _and list 2 with length *l*_2 _have *m *overlapping genes, then the score from list 1 to list 2 is POG_12 _= *m*/*l*_1 _and the score from list 2 to list 1 is POG_21 _= *m*/*l*_2_. To reduce the effect of list lengths on the POG scores, we also calculated the normalized scores as follows [33]:(1)

where *E*(*POG*_12_) and *E*(*POG*_21_) are the POG scores expected by random chance, which are estimated separately as the average of the scores for 10 000 pairs of gene lists (with length *l*_1 _and *l*_2_) extracted randomly from the human genome.

### Evaluating the functional consistency of gene lists at the network level

We proposed to evaluate the functional consistency of two gene lists by taking into account functionally similar genes between the lists. First, a gene was defined to be functionally similar to a gene list if its PPI links to the genes in the list were significantly more than expected by random chance (*P *< 0.05). Here, a PPI link between two genes means that the two genes interact with each other or share at least one neighbour in the PPI network [35-37]. Suppose a gene has *k *PPI links to the *M *genes in a list, then the probability of observing at least *k *links by random chance can be calculated by the hypergeometric probability model:(3)

where *N *is the number of all the possible links between this gene and other genes in the PPI network, and *n *is the observed number.

Then, we proposed the POGF score between gene list 1 with length *l*_1 _and list 2 with length *l*_2 _as follows:(4)

where *O *is the number of genes shared by the two lists, and *Of*_12 _(or *Of*_21_) is the number of genes in list 1 (or list 2) not shared by but functionally similar to genes in list 2 (or list 1).

To remove the effect of list lengths, we normalized the POGF scores for the two lists as follows [33]:(6)

where *E*(*POGF*_12_) and *E*(*POGF*_21_) are the scores expected by random chance for two gene lists (with length *l*_1 _and *l*_2_), which are estimated separately as the average of the scores of 10 000 pairs of gene lists (with length *l*_1 _and *l*_2_) extracted randomly from all the genes in the PPI network.

### Statistical significance of a consistency score

To evaluate the significance of an observed POG (or POGF) score between two lists (with length *l*_1 _and *l*_2_), we selected randomly a pair of gene lists (with length *l*_1 _and *l*_2_) and calculated the score by the same method. This process was repeated 10 000 times. The significance (*P *value) of the score was calculated as the percentage of the random scores that were larger than the observed score. The *P *value of a nPOG or nPOGF score is the same as that of the corresponding POG or POGF score because the E(POG) or E(POGF) that is used to normalize the POG or POGF score is a constant [33].

### Selecting functionally consistent cancer genes

The CGC database comprises cancer genes with relatively stringent criteria. Therefore, we filtered other gene lists according to their functional similarity to the genes included in the CGC database. A gene was selected if its functional links to genes from CGC were significantly more than expected by random chance, with the *P *value calculated by formula (3) and corrected by the FDR control [38]. Then, for the selected genes and the remaining ones, respectively, we calculated the probabilities of their enrichment in each of the 10 cancer pathways described in the Cancer Cell Map database [39], by the hypergeometric distribution model.

## Results

### Consistency between gene lists in terms of gene overlapping

**Figure 1:**
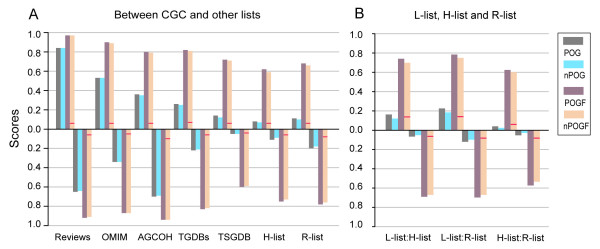
**The consistency scores between different cancer gene lists**. A. The bars above the x-axis depict POG (nPOG) and POGF (nPOGF) scores from other lists to CGC, and the bars below the x-axis depict the POG (nPOG) and POGF (nPOGF) scores from CGC to other lists. The red lines within the bars depict the POGF scores expected by random chance. B. The POG (nPOG) and POGF (nPOGF) scores between the H-list, L-list and R-List. The bars above the x-axis depict POG (nPOG) and POGF (nPOGF) scores from H-list to L-list, from L-list to R-list and from H-list to R-list respectively. The bars below the x-axis depict scores from L-list to H-list, from R-list to L-list and from R-list to H-list respectively. The red lines within the bars depict the POGF scores expected by random chance.

CGC is the most widely utilized cancer gene data source in various applications [3-6,8,11,22,23], therefore, we used it as a baseline for the comparison. The POG (nPOG) score from Reviews to CGC was 0.84 (0.84) and 0.65 (0.64) from CGC to Reviews. However, as shown in Figure 1A, most other gene lists were highly inconsistent with CGC. The POG (nPOG) score from OMIM to CGC was 0.53 (0.53) and 0.34 (0.34) in the other direction. Notably, 55% of the genes in CGC were labelled with leukaemia/lymphoma, whereas only about 21% of the genes in OMIM were associated with this cancer. The POG (nPOG) score from AGCOH to CGC was 0.36 (0.35) and 0.70 (0.69) from CGC to AGCOH. Among the 263 genes shared by these two databases, 152 (60%) were associated with haematological cancer. The POG (nPOG) score from TGDBs to CGC was 0.26 (0.25) and 0.22 (0.21) from CGC to TGDBs, which only included genes discovered in six epithelial cancer types. TSGDB only included tumor suppressor genes, therefore, the POG (nPOG) score from it to CGC was only 0.14 (0.12) and 0.05 (0.05) in the other direction.

The above results showed that these lists of cancer genes were highly inconsistent in terms of gene overlapping. However, all the observed POG and nPOG scores were significantly larger than the scores expected by random chance (*P *< 1.0 E-04).

### Functional consistency between gene lists

**Figure 2:**
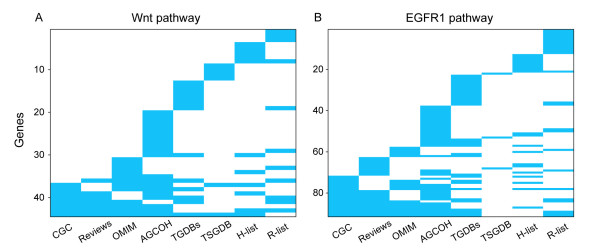
**Distribution of cancer genes in the Wnt and EGFR1 pathways**. The x axis depicts the eight cancer gene lists and the y axis depicts the overlapping genes between the genes in the eight lists and all the genes in the pathways.

Although different gene lists are inconsistent in terms of overlapping genes, they could each capture different genes in the same pathways associated with cancer. For example, as shown in Figure 2, different lists of cancer genes covered various genes in the Wnt and EGRF1 pathways. Next, we evaluated the functional consistency of the gene lists based on the POGF (nPOGF) scores. As shown in Figure 1A (bars above the x axis), the POGF (nPOGF) scores from Reviews, OMIM, AGCOH, TGDBs and TSGDB to CGC were 0.97 (0.97), 0.90 (0.89), 0.80 (0.79), 0.82 (0.81) and 0.72 (0.71), respectively. In another direction, the POGF (nPOGF) scores from CGC to the other lists were as high as 0.92 (0.91), 0.87 (0.87), 0.94 (0.94), 0.83 (0.82) and 0.60 (0.59). From TGDBs to CGC, although the POG (nPOG) score was only 0.26 (0.25), the POGF (nPOGF) score was as high as 0.82 (0.81), which indicated that the genes in TGDBs shared similar functions with those in CGC. Another impressive result was that the POGF (nPOGF) score from TSGDB to CGC was as high as 0.72 (0.71) and 0.60 (0.59) in the other direction, although the corresponding POG (nPOG) scores were very low.

All the observed POGF and nPOGF scores were statistically significant (*P *< 1.0 E-04).

### **Consistency of gene lists discovered in low- and high-throughput studies**

We used the L-list for the 1208 distinct genes extracted from the six databases that contained cancer genes discovered in small-scale studies, and the H-list for the 491 cancer genes identified by mutational screening for four cancer types [19,20,22,23]. From the L-list to the H-list, the POG (nPOG) score was as low as 0.07 (0.05), which indicated that most cancer genes accumulated in the small-scale studies were not rediscovered in the high-throughput data. From the H-list to the L-list, the score was a little larger, but still low, at 0.16 (0.12), which indicated that the high-throughput screening studies could find only a small fraction of all cancer genes. For each of the four cancer types, the consistency of the sub-lists of cancer genes extracted from the L-list and H-list was also very low (Table 2).

**Table 2:** The scores between sub-lists of cancer genes for each cancer type

Tumor	Gene No. (L:H)	From L to H				From H to L			
		
		POG	nPOG	POGF	nPOGF	POG	nPOG	POGF	nPOGF
Breast	67:122 ^a^	0.11	0.10	0.62	0.60	0.04	0.04	0.58	0.56
Colon	44:118	0.24	0.23	0.82	0.81	0.06	0.06	0.64	0.63
Pancreatic	13:44	0.36	0.35	0.62	0.60	0.10	0.10	0.34	0.32
Glioblastoma	18:50	0.37	0.37	0.83	0.83	0.10	0.10	0.64	0.63

Note: ^a ^the number of cancer genes with PPI data for the low- and high-throughput data. All the scores are statistically significant (*p *values < 1.0 E-04).

On the other hand, the POGF (nPOGF) score from the L-list to the H-list was 0.69 (0.67) and 0.74 (0.70) in the other direction. Thus, functionally, cancer genes found in small-scale experiments were consistent with those found in the high-throughput studies. As shown in Table 2, from the sub-lists of cancer genes discovered by the genome screening to the sub-lists of cancer genes discovered in small-scale experiments for breast, colon and pancreatic cancers, and glioblastoma, the POGF (nPOGF) scores were as high as 0.62 (0.60), 0.82 (0.81), 0.62 (0.60) and 0.83 (0.83), respectively. In the other direction, the POGF (nPOGF) scores were much lower, which were 0.58 (0.56), 0.64 (0.63), 0.34 (0.32) and 0.64 (0.63) for the four cancer types, respectively. Thus, for each cancer type, the cancer genes discovered by the genome screening might cover more functions of cancer genomes than the cancer genes accumulated from small-scale experiments.

We used the R-list for the 645 cancer genes indentified by the high-throughput retroviral insertional mutagenesis screening. As shown in Figure 2B, the POG (nPOG) scores from the R-list to the L-list were 0.12 (0.10) and 0.22 (0.18) in the other direction. However, the POGF (nPOGF) scores were as high as 0.70 (0.68) and 0.78 (0.75) in the two directions, respectively. These results were similar to those for the H-list. The POG (nPOG) score from the R-list to the H-list was only 0.05 (0.03) and 0.04 (0.02) in the other direction. The POGF (nPOGF) scores in the two directions were 0.57 (0.53) and 0.62 (0.60), respectively, which suggested that these two lists of cancer genes were less functionally overlapped.

### Cancer genes selected by functional consistency and the F-Census database

Even at the functional level, some inconsistency still existed between CGC and other databases. Therefore, we selected genes from other lists according to their functional similarity to genes in the CGC database. With FDR 1% and FDR 5%, respectively, 685 and 756 genes were selected. As shown in Table 3, in most of the 10 cancer-related pathways from Cancer Cell Map, the selected genes were significantly enriched (*P *< 0.01), whereas the remaining genes were not (*P *> 0.01). In these pathways, most ratios of the selected genes to the other genes were >10, which supports the hypothesis that the selected genes are more likely to be cancer-associated.

**Table 3:** The enrichment of the selected genes in cancer pathways (FDR < 0.01)

Signal pathway names	*p *values*	*p *values**	Ratios
Alpha6Beta4Integrin_pathway(54^a^)	4.75E-12	0.89	24
AndrogenReceptor_pathway(103)	2.76E-11	1	Inf***
EGFR1_pathway(179)	0	0.64	10.7
Hedgehog_pathway(23)	3.41E-05	0.01	1.75
ID_pathway(25)	1.30E-10	1	Inf
KitReceptor_pathway(69)	0	0.72	16.5
NOTCH_pathway(80)	9.40E-11	0.33	5
TGFBR_pathway(159)	8.29E-13	0.69	9.8
TNFAlphaNFkb_pathway(189)	0	0.82	7.2
Wnt_pathway(105)	0	0.01	2.6

Note: ^a ^the number of genes in the pathway;* the *p *value of the enrichment of the selected genes; ** the *p *value of the enrichment of the rest cancer genes; *** Inf means none of the rest genes are annotated in the pathway.

Based on the above results, we have developed a database named F-Census for extracting functionally consistent cancer genes from different data sources. This database is available at http://bioinfo.hrbmu.edu.cn/fcensus/. Using this database, users can extract cancer genes from several databases to obtain their union and intersection gene sets, thus providing information about cancer genes, such as their type (oncogenes and tumor suppressor genes), their occurrence in different cancers, and their mutation frequencies estimated from the high-throughput studies. Also, the users can obtain the cancer gene list pre-selected by our criteria based on their functional similarity to genes in CGC. The users can upload a list of candidate genes and prioritize the genes in the list according to their functional similarity to cancer genes in CGC. Finally, the users can look up the functional categories enriched with cancer genes from various cancer gene lists (please see the Help page on our website for details).

## Discussion

In this study, we showed that current cancer gene data sources were highly inconsistent in terms of gene overlapping. This suggested that the sample sizes used in either the small-scale studies or high-throughput genome screening might be too small to provide enough power for consistently capturing genes causally related to the extremely heterogeneous cancer [1,12,40,41]. Nevertheless, most cancer gene lists were functionally consistent, which indicated that they might all come from some key pathways associated with cancer. Based on this assumption, for a list of cancer genes, there should be subsets of non-redundant genes that could functionally represent the full list of genes. Actually, by the algorithm described in additional file 1, we could select 75 genes from GCG, which could represent all the 377 cancer genes from CGC, in the sense that all 377 cancer genes are frequently connected to the 75 cancer genes in the PPI network (POGF score = 1). A future study is warranted to establish whether such a non-redundant subset of genes hints at the organization of cancer-related functions.

The biological function of a gene can be defined at several levels, ranging from the basic biological attributes of a protein product, to the nature of physical and regulatory interactions, membership in a given biological pathway, and membership of a specific biological network (such as a PPI sub-network) [10,11]. We could consider that the functional consistency of gene lists evaluated by the POGF score based on PPI links is at the PPI network level. We could also evaluate the consistency of gene lists at other functional levels. For example, using GO terms at separate levels of the GO hierarchy, we could evaluate the consistency of gene lists at various levels of pathway specificity, and find the most specific level at which the consistency changes from high to low. To design such GO-based consistency scores, we need to consider the limitations that GO levels are artificially defined, and a large fraction of genes are only annotated to general high-level terms.

It would be interesting to identify a functional level at which cancer genes of the same cancer type overlap strongly and cancer genes of different cancer types can be distinguished. However, it might be difficult, if not impossible, to achieve this goal because most genes responsible for tumorigenesis of different cancer types might disrupt the same or similar pathways [29]. In the KEGG database, all the 14 pathways labelled with cancer types, according to some so far agreed cancer-type-specific genes, such as APC of colorectal cancer, actually consist of similar biological pathways, such as mitogen-activated protein kinase, p53, transforming growth factor-β and Jak-Stat pathways [42]. Statistically, because of the small samples studied for some cancers, the lists of cancer genes accumulated so far for different cancers might be inconsistent and insufficient for functional discrimination of cancer types. As demonstrated in our previous work [34], even for the same cancer, the true disease markers identified in different studies with insufficient samples (and thus low statistical power) are highly likely to be inconsistent. We believe that it might be necessary to use more samples and combine functional data with tissue expression data to study cancer-type-specific mechanisms.

The literature-based interaction data in the HPRD database might be biased towards well-studied cancer genes. However, Ciccarelli *et al*. [6] have argued that such a bias might be ignorable because, in the high-throughput PPI data, cancer genes also tend to have higher degrees in the PPI network than other genes. Similarly, using cancer genes with both literature-based interaction data and high-throughput interaction data in the HPRD database, we found that the literature-based degrees of these cancer genes were significantly correlated with their high-throughput data-based degrees (*r *= 0.4, *P *< 0.01, Spearman's rank correlation), indicating our functional assessment would not be severely affected by the research bias. This problem should be further addressed when more high-throughput PPI data become available. Another concern that should be addressed is that current PPI data are incomplete. However, as in the present study, the functional similarity measure based on indirect PPI links might lessen the effect of the incompleteness of the direct PPI links.

In our study, CGC was employed as a benchmark for the comparison because it is the most widely applied data source. However, this benchmark might be biased because genes collected in CGC tend to originate from lymphoma/leukaemia, and most genes were of translocation mutations. Thus, in our future work, we will exploit other criteria to define more reliable and unbiased benchmark cancer gene sets. One approach might be to find genes non-randomly co-mutated with other genes in cancer samples. As implied by our work [43] and Yeang *et al*. [44], this statistically sound approach could bypass the unsolved difficulty of the background mutation rate estimation in so-far used prediction methods.

Finally, we note that the F-Census database is still under development, and is aimed at including more comprehensive information on cancer genes. For example, we have included in the database genes non-randomly co-mutated with other genes in cancer samples, which can provide strong statistical evidence on their involvement and functional coordination in cancer [9,44]. Additionally, we have collected miRNAs that could play important roles in oncogenesis by regulating cancer genes [45-47]. We will also try to consider the full spectrum of genetic and epigenetic changes in cancer in our future studies [48,49].

## Conclusions

Because cancer is an extremely heterogeneous disease, low consistency in the discovery of cancer genes could have been expected in studies that have used insufficient samples. Although most data sources have low gene overlapping, they are highly consistent at the functional level, which indicates that they might capture separately different genes in a few key pathways associated with cancer. Our database provides biologists with a useful tool for browsing and extracting functionally consistent cancer genes from various data sources.

## Authors' contributions

XG and ZG designed the project and contributed to the draft of the manuscript. XG, RW, YZ, WZ carried out all the data analysis and result interpretation. RW and LC developed the database. YG, LZ, JW and JZ contributed to the draft of the manuscript.

## Supplementary Material

Additional file 1 Algorithm for finding a non-redundant gene set from a list. Click here for file
