# Supplementary material for: Extracting consistent knowledge from highly inconsistent cancer gene data sources
Source: BMC Bioinformatics. 2010 Feb 5;11:76. doi: 10.1186/1471-2105-11-76 (PMC2832783; doi:10.1186/1471-2105-11-76)
Supplement: Additional file 1 — Algorithm for finding a non-redundant gene set from a list. [file 1471-2105-11-76-S1.DOC]

## Searching the smallest gene sets to represent cancer-related functions

Given the heterogeneity nature of cancer, different cancer genes found in different cancer samples (or studies) may come from only a few cancer-related functions. Based on this assumption, we designed an empirical algorithm to select the smallest sub-lists of genes which could functionally represent most genes in the whole list. Their sizes could provide an estimation of the number of functionally non-redundant (or independent) genes in the whole list.

Starting from the entire genes list consisting of genes for a cancer type, we iteratively removed one gene at a step and calculated the POGF score between the remaining genes and the entire gene list. In each step, we selected a sub-list with the largest POGF score as the input of the next iteration and meanwhile deleted the corresponding gene. When there were multiple sub-lists with the same largest POGF score, we empirically deleted the gene with the smallest degree in the PPI network which may contribute less to the POGF score in the following procedures. This process was iteratively repeated in the selected sub-list till the POGF score between any of its sub-list and the entire list was below a given threshold such as 1.
